# Studies on the Phytochemical Profile of *Ocimum basilicum* var. *minimum* (L.) Alef. Essential Oil, Its Larvicidal Activity and In Silico Interaction with Acetylcholinesterase against *Aedes aegypti* (Diptera: Culicidae)

**DOI:** 10.3390/ijms231911172

**Published:** 2022-09-22

**Authors:** Anderson de Santana Botelho, Oberdan Oliveira Ferreira, Mozaniel Santana de Oliveira, Jorddy Neves Cruz, Sandro Henrique dos Reis Chaves, Alejandro Ferraz do Prado, Lidiane Diniz do Nascimento, Geilson Alcantara da Silva, Cristine Bastos do Amarante, Eloisa Helena de Aguiar Andrade

**Affiliations:** 1 Faculty of Chemistry, Institute of Exact and Natural Sciences, Federal University of Pará, Augusto Corrêa Street, S/N, Guamá, Belém 66075-900, Pará, Brazil; 2 Adolpho Ducke Laboratory—Botany Coordination, Emílio Goeldi Museum of Pará, Perimetral Avenue, 1901, Terra Firme, Belém 66077-830, Pará, Brazil; 3 Laboratory of Functional and Structural Biology, Institute of Biological Sciences, Federal University of Pará, Belém 66075-110, Pará, Brazil; 4 Institute of Biological Sciences, Federal University of Pará, Augusto Corrêa Street, S/N, Guamá, Belém 66075-900, Pará, Brazil; 5 Chemical Analysis Laboratory—Coordination of Earth Sciences and Ecology, Emílio Goeldi Museum of Pará, Perimetral Avenue, 1901, Terra Firme, Belém 66077-830, Pará, Brazil

**Keywords:** natural products, *Ocimum*, larvae, mosquito, acetylcholinesterase, ecological

## Abstract

*Aedes aegypti* L. (Diptera: Culicidae) is an important transmitter of diseases in tropical countries and controlling the larvae of this mosquito helps to reduce cases of diseases such as dengue, zika and chikungunya. Thus, the present study aimed to evaluate the larvicidal potential of the essential oil (EO) of *Ocimum basilicum* var. *minimum* (L.) Alef. The EO was extracted by stem distillation and the chemical composition was characterized by gas chromatography coupled with mass spectrometry (GC/MS and GC-FID). The larvicidal activity of EO was evaluated against third instar *Ae. aegypti* following World Health Organization (WHO) standard protocol and the interaction of the major compounds with the acetylcholinesterase (AChE) was evaluated by molecular docking. The predominant class was oxygenated monoterpenes with a concentration of 81.69% and the major compounds were limonene (9.5%), 1,8-cineole (14.23%), linalool (24.51%) and methyl chavicol (37.41%). The *O. basilicum* var. *minimum* EO showed unprecedented activity against third instar *Ae. aegypti* larvae at a dose-dependent relationship with LC_50_ of 69.91 (µg/mL) and LC_90_ of 200.62 (µg/mL), and the major compounds were able to interact with AChE in the Molecular Docking assay, indicating an ecological alternative for mosquito larvae control.

## 1. Introduction

*Aedes aegypti* L. (Diptera: Culicidae) is the vector responsible for the transmission of infectious diseases such as dengue, yellow fever, zika and chikungunya and has caused serious public health problems, especially in tropical regions [1]; these problems are related to the accelerated rate of proliferation of this mosquito, which has strong resistance to insecticides and commercial repellents. Thus, controlling the proliferation of this vector remains the main tool to eradicate or reduce the harmful effects of *Ae. aegypti* [2].

Natural products from plants are promising in controlling this proliferation due to their biological properties, as well as essential oils, which are defined as volatile substances of natural origin and diversified chemical composition [3,4]. The chemical nature of these volatile compounds consists mainly of molecules with low molecular weight that may be related to biological activities in essential oils with antimicrobial, anti-inflammatory and larvicidal effects; it is important to mention that essential oils have gained prominence as an alternative source in the control and combat of vectors such as *Ae. aegypti* [5,6].

There are studies regarding the Lamiaceae family that prove the effectiveness of essential oils with larvicidal potential against some vectors, as in the study [7], in which the essential oil of *Ocimum campechianum* Mill. showed high larvicidal effects against *Ae. aegypti*. In the study carried out [8], testing the essential oil of *Thymus vulgaris* L. and *Origanum majorana* L. against larvae of *Anopheles labranchiae* (Diptera: Culicidae), both essential oils had strong larvicidal potential. Likewise, the essential oil of *Origanum vulgare* L. showed activity against *Anopheles stephensi*, *Anopheles subpictus*, *Culex quinquefasciatus* and *Culex tritaeniorhynchus*. In addition, it is important to know the molecular interaction mechanisms, through in silico studies of compounds present in essential oils with potential larvicidal activity, this may be a viable strategy for the development of new technical to combat mosquito larvae that are vectors of tropical diseases and authors have reported that acetylcholinesterase (AChE) is an important molecular target for understanding the larvicidal activity [9,10], demonstrated by several authors in studies of the larvicidal potential of essential oils from different plants [11,12,13,14].

In this context, *Ocimum basilicum* var. *minimum* (L.) Alef. may be a source of bioactive compounds with larvicidal potential [15], is a species of herb or shrub belonging to the Lamiaceae family, terrestrial type, popularly known as Bush Basil. Regarding the composition of the essential oils of *O. basilicum* var. *minimum*, studies report the presence of the monoterpenic compound linalool as one of the main compounds [16,17]. In another study, the essential oil of *O. basilicum* var. *minimum* was characterized by the major compounds linalool (52.7%), eugenol (9.1%) and bornyl acetate (1.9%), while in another species of the same genus, the methyl eugenol (78.02%), α-cubebene (6.17%) and nerol (0.83%) compounds characterized the chemical profile of *O. basilicum* essential oil [18].

Therefore, the objective of this study was to carry out the chemical characterization of *O. basilicum* var. *minimum* essential oil collected in the Brazilian Amazon and to evaluate the larvicidal potential against third instar larvae of *Ae. aegypti* for application in the control of diseases transmitted by this mosquito.

## 2. Results and Discussion

### 2.1. Essential Oil Yield

The essential oil (EO) of *O. basilicum* var. *minimum* leaves showed a yield of 1.57% (*v*/*w*); this yield was higher than that found [19] in essential oils extracted from the dry leaves of *O. basilicum* var. *minimum* by hydrodistillation, which showed yields of 1.20% (*v*/*w*), 1.06% (*v*/*w*) and 1.0% (*v*/*w*), respectively, in the years 2005, 2007 and 2008, and lower when compared to the same study in the years 2003, 2004 and 2006, which showed yields of 1.93% (*v*/*w*), 1.83% (*v*/*w*) and 1.78% (*v*/*w*). In another study carried out by Safari Dolatabad et al. [20], the essential oil of *O. basilicum* var. *minimum* showed a yield of 0.5% (*v*/*w*), much lower than that found in this study.

### 2.2. Chemical Composition

**Figure 1:**
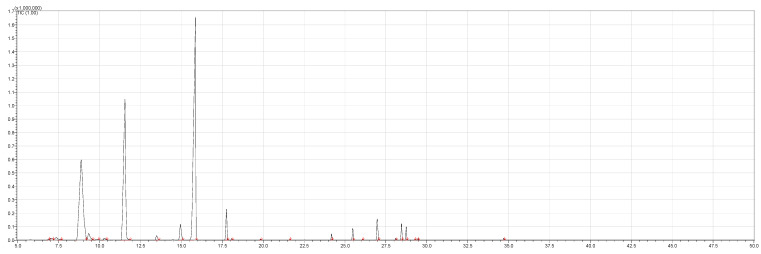
Ion-chromatogram of essential oil of *O. basilicum* var. *minimum*.

Table 1 presents the data related to the chemical composition obtained by GC-MS in ascending order of retention rates for each constituent, with 24 constituents identified in the EO of *O. basilicum* var. *minimum*, representing 99.65% of the oil composition. The chemical profile of the EO was characterized by the major compounds methyl chavicol (37.41%), linalool (24.51%), 1,8-cineole (14.23%) and limonene (9.50%). The levels of methyl chavicol and linalool were higher than that found by [21] in a study carried out in South Africa with the essential oil of *O. basilicum* var. *minimum*, with methyl chavicol contents of 34.3% and linalool of 17.8%. Linalool (25.6%) has also characterized the essential oil of *O. basilicum* var. *minimum*, as well as the compound geranyl acetate (45.6%). The chemical composition of the EO of this species in collections carried out in Iran, during the years 2003 to 2008, demonstrated the strong presence of linalool with concentrations ranging from 40.2% to 88.34%, followed by 1,8-cineole (1.46% to 8.87%) and eugenol (0.28% to 7.23%) [19]. Ion-chromatogram of the essential oil is shown in Figure 1.

Essential oils from species of the genus *Ocimum* have presented both linalool and methyl chavicol; these compounds have characterized the essential oils of *O. basilicum*, *O. americanum*, *O. campechianum*, *O. kilimandscharicum* [23], *O. gratissimum* and *O. tenuiflorum* [24]. Furthermore, studies report antioxidant activities that may be related to the presence of linalool and methyl chavicol [25]. Linalool is described as having cytotoxic activities against HeLa, HEp-2 and NIH 3T3 type cancer cells [26], and antibacterial activities against *Listeria monocytogenes*, *Enterobacter aerogenes*, *Escherichia coli* and *Pseudomonas aeruginosa* [27]. Methyl chavicol is reported for antimicrobial action in essential oils and against phytopathogenic agents such as *Brenneria nigrifluens* [28]. Studies in silico have indicated that this compound has antilipase biological action and may be a promising molecular target for the treatment of diseases related to oxidative damage [29].

The monoterpenic compounds 1,8-cineole and limonene showed significant levels in the composition of the essential oil of the species under study; these contents were higher than those found in the essential oil of a sample collected in Gabon, Africa, which were 0.7% and 0.2%, respectively. Other species of the genus *Ocimum* of the aforementioned study were also characterized by the respective compounds, as described in the essential oil from *O. basilicum*, *O. gratissimum*, *O. americanum* and *O. lamiifolium* [21]. There are reports showing that 1,8-cineole has biological properties with antibacterial, antifungal, anesthetic and allelopathic potential [30].

Limonene naturally presents two enantiomeric forms: R-(+)- and S-(−)-. Among these forms, the terpene R-(+)- limonene is the most common found in nature; this compound has shown potential in activities with fumigant and repellent action, electroencephalographic, cytotoxicity against tumor cells and antimicrobial activities against bacteria [31,32].

### 2.3. Larvicidal Activity

Mortality data for third instar *Ae. aegypti* larvae exposed to different concentrations of *O. basilicum* var. *minimum* essential oil. demonstrate the efficacy of the larvicidal action on mosquito larvae at a dose-dependent relationship with a LC_50_ value of 69.91 µg/mL (CI = 61.89–78.58 µg/mL) and a LC_90_ value of 200.62 µg/mL (CI = 179.45–227.84 µg/mL). There was no significant difference in mortality between the control groups of larvae exposed to water and 2% DMSO, indicating that there is no influence of the diluent on the mortality observed in the solutions of EO (*p* < 0.05).

The World Health Organization (WHO) does not establish a criterion for evaluating the larvicidal potential of natural products, but some authors consider LC_50_ values between 50 and 100 µg/mL as active [33], moderately active [34] or with significant activity [35], framing the EO of *O. basilicum* var. *minimum* in these categories (LC_50_ = 69.91 µg/mL); this activity can be attributed mainly to the major compounds present in the EO, or even the synergism between them and the other components. For instance, methyl chavicol and limonene have already been shown to be highly active against third instar larvae of *Ae. aegypti* with LC_50_ values of 46.40 µg/mL (CI = 42.50–50.00 µg/mL) and 13.0 µg/mL (CI = 10.50–16.70 µg/mL), respectively [36].

These results are in line with those obtained for most species of the Lamiaceae family that represent 10.5% of the active oils (LC_50_ < 100 µg/mL) against third instar larvae of *Ae. aegypti*, behind only the Myrtaceae family with 13.5% [37]. Within the genus *Ocimum*, the EO of *O. basilicum* var. *minimum* showed higher activity than the EOs of *O*. *basilicum, O*. *sanctun*, *O*. *campechianum* and *O*. *carnosum*, but showed lower activity than the EOs of *O*. *suave*, *O. americanum* and *O*. *gratissimum* (Table 2); these data reveal the similarity of the larvicidal activity of the EO of *O. basilicum* var. *minimum* with other species of the genus, mainly due to the presence of common constituents such as limonene, methyl chavicol, linalool and 1,8-cineole.

The larvicidal activity observed for the EO was lower when compared to synthetic larvicides [43]; this lower efficiency and the higher cost compared to synthetic compounds is found for the vast majority of plant derivatives and makes their use much lower than conventional larvicides [37]. suHowever, the increasing use of synthetic larvicides has led to an increase in the resistance of these organisms [44]. Thus, the EO of *O. basilicum* var. *minimum* becomes a relevant alternative to combat *A. aegypti* larvae or even to obtain highly active substances (LC_50_ < 50.00 µg/mL) such as methyl chavicol and limonene.

### 2.4. Evaluation of the Interaction of EO Compounds with AChE

**Figure 2:**
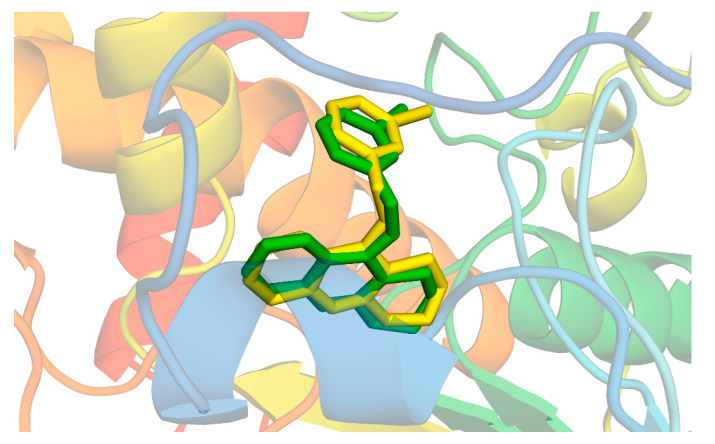
Superposition of crystallographic (green) and redocked (yellow) ligands.

Molecular modeling approaches have been successfully used to evaluate the interaction of major volatile compounds with molecular targets of pharmacological interest [45,46,47]. We used AChE as a molecular target because this enzyme is a promising molecular target for essential oils with larvicidal activity [9]. Before starting the docking, MD simulations and free energy analyzes, the crystallographic ligand was redocked. Redocking was performed to assess whether the software was capable of simulating the experimental binding mode found in the crystallographic of the *AChE*-tacrine derivative complex 9-(3-iodobenzylamino)-1,2,3,4-tetrahydroacridine. According to the literature, for the docking protocol used to be considered satisfactory, the RMSD found between the crystallographic ligand and the redocked ligand must be equal to or less than 2 angstroms [48,49,50]. The results of this study showed an RMSD value equal to 1.8 angstroms; thus, our docking protocol is suitable to evaluate the way of interaction of biomolecules with the *AChE* binding cavity. Figure 2 shows the overlap between crystallographic and redocked ligands.

**Figure 3:**
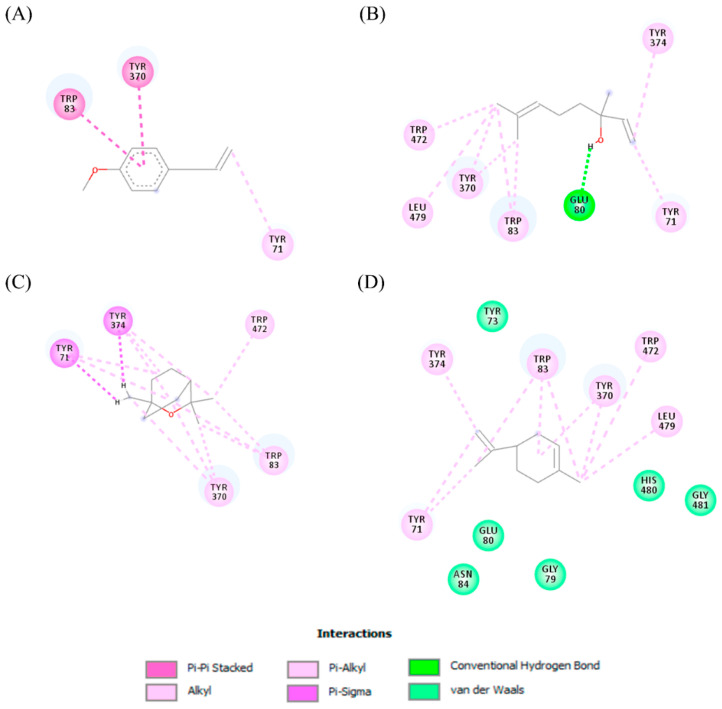
Molecular interactions established between (**A**) methyl chavicol, (**B**) linalool, (**C**) 1,8-cineole and (**D**) limonene with AChE pocket binding.

Docking analyzes have been used to evaluate the molecular binding mode of compounds present in EO. Through these analyzes, it is possible to evaluate the interactions established between the EO compounds and the molecular target under study. In Figure 3, it is possible to visualize the interactions and the chemical nature of the bonds formed between the compounds present in the EO and the AChE.

**Figure 4:**
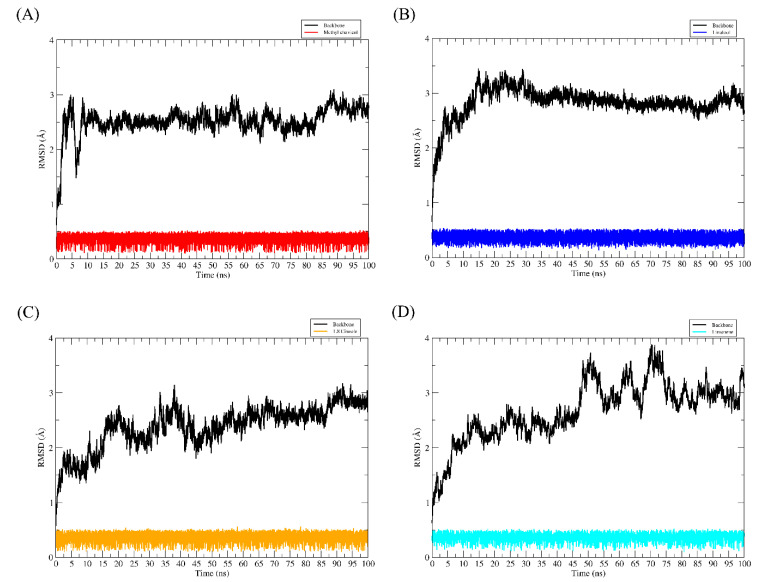
RMSD of AChE-ligand complexes (**A**–**D**).

In the enzyme binding pocket, all ligands remained interacting throughout the simulation. During the 100 ns of MD simulations, the ligands remained bound to the enzyme and exhibited a low fluctuation profile, as can be seen in Figure 4A–D.

Methyl chavicol interacted with the amino acid residues of Trp83 and Tyr370 through stacked π-π interactions formed mainly by the benzene ring of the molecule. Linalool remained bound to the active site of the protein, forming mainly alkyl-type interactions with residues of Trp427, Leu479, Tyr370, Trp83, Tyr71 and 374 and a hydrogen bond with Glu80. The 1,8-cineole interacted with Trp472, Trp83 and Tyr370 through alkyl and π-alkyl hydrophobic interactions and with the residues of Try374 and Tyr71 π-σ interactions. Limonene has formed van der Waals interactions with Asn84, Glu80, Gly79, His480, Gly481 and Tyr73 and alkyl or π-alkyl interactions with Tyr71, Tyr374, Trp83, Tyr370, Trp472 and Leu479.

**Figure 5:**
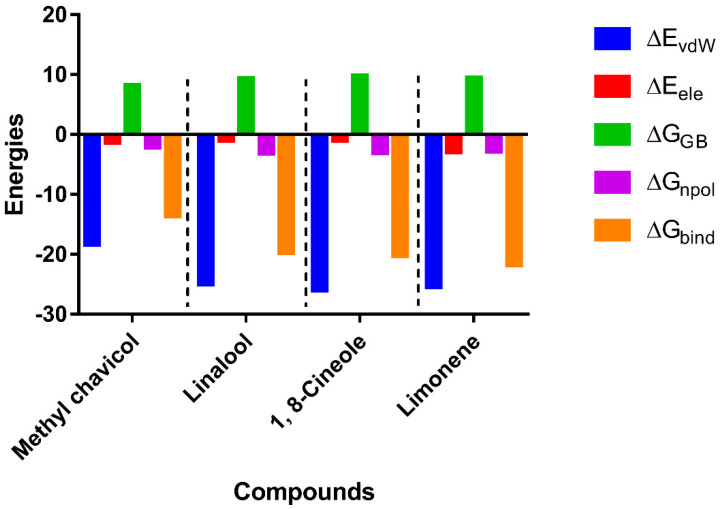
Binding energy values and energy components. ΔE_vdW_, contributions by van der Waals interactions; ΔE_ele_, electrostatic energy; ΔG_GB_, polar solvation energy; ΔG_npol_, nonpolar solvation energy; ΔG_bind_, binding affinity.

As observed in the MM/GBSA results (Figure 5), the complexes were spontaneously established, since the ligands (A) methyl chavicol, (B) linalool, (C) 1,8-cineole and (D) limonene obtained affinity energy of −13.65, −19.73, −20.29 and −21.80 kcal/mol, respectively.

## 3. Materials and Methods

### 3.1. Plant Material

Leaves of *O. basilicum* var. *minimum* were collected in the municipality of Ananindeua, Pará, Brazil. The specimen of the sample was identified and incorporated into the collection of the João Murça Pires Herbarium (MG) of the Emílio Goeldi Museum of Pará, in the collection of Aromatic Plants of the Amazon, Belém, Pará, under the registration MG167656.

### 3.2. Preparation of Botanical Material

The samples of *O. basilicum* var. *minimum* leaves were dried in an oven with air circulation at a temperature of 35 °C for 5 days and then ground in a knife mill (Tecnal, model TE-631/3, Brazil). The moisture content was analyzed using an infrared moisture analyzer (ID50; GEHAKA, Duquesa de Góias, Real Parque, São Paulo, Brazil), in the temperature range from 60 to 180 °C with increments of 1 °C and bidirectional output.

### 3.3. Essential Oil Extraction

The essential oil (EO) from the leaves of *O. basilicum* var. *minimum* was extracted using 130 g of dried material by stem distillation with a Clevenger-type apparatus for 3 h as described by Oliveira et al. [51]. The EO obtained was dehydrated with anhydrous sodium sulfate and centrifuged for 5 min at 3000 rpm.

### 3.4. Essential Oil Analysis

The chemical compositions of the EO of *O. basilicum* var. *minimum* were analyzed as reported by our research group [52,53], using a Shimadzu QP-2010 (Kyoto, Japan) plus gas chromatography system equipped with an Rtx-5MS capillary column (Restek Corporation, Bellefonte, PA, USA) (30 m × 0.25 mm; 0.25 µm film thickness) coupled with a mass spectrometer (GC/MS) (Shimadzu, Kyoto, Japan) and the components were quantified using gas chromatography (CG) on a Shimadzu QP-2010 system (Kyoto, Japan), equipped with a flame ionization detector (FID). The program temperature and injection were the same operating conditions as described in the literature [54,55], except for the carrier hydrogen gas, under the same operating conditions as before. The retention index for all volatile constituents was calculated using a homologous series of n-alkanes (C_8_–C_40_) Sigma-Aldrich (San Luis, CA, USA), according to van den Dool and Kratz [56]. The components were identified by comparison of: (i) the experimental mass spectra with those compiled in libraries, and (ii) their retention indices to those found in the literature [57].

### 3.5. Larvicidal Assay

The methodology adopted was the World Health Organization standard protocol [58]. To obtain the larvae, *Ae. aegypti* eggs were placed in a tray with 500 mL of distilled water added to 1 g of rat chow. Hatching occurred within 24 h and larvae were allowed to grow. Upon reaching the third instar, batches of 25 larvae were transferred by droppers to small disposable test cups, each containing 100 mL of water, and added appropriate volume of stock solution to obtain the desired target dosage.

The larvae were exposed to concentrations of 15.62, 31.25, 62.5, 125, 250 and 500 ppm (*v*/*v*) of the oil diluted in 2% dimethyl sulfoxide aqueous solution (DMSO 2%) for 24 h. The entire assay was performed in quadruplicate, with 25 larvae for each concentration, at a temperature of 25 °C and a photoperiod of 12 h of light followed by 12 h in the dark. Negative controls water (H_2_O) and DSMO 2% were evaluated under the same conditions as the sample.

### 3.6. In Silico Analysis

#### 3.6.1. Molecular Docking

The compounds methyl chavicol, linalool, 1,8-cineole and limonene were drawn in GaussView 6 and their structure were optimized via B3LYP/6-31G* using the Gaussian quantum chemistry software 16 [59]. The software Molegro Virtual Docker [60] was then used to assess how these compounds are able to interact with the acetylcholinesterase (*AChE*) binding cavity. For that, the crystal structure of the protein was collected in the Protein Data Bank (www.rcsb.org, accessed on 5 March 2022) and located using the PDB ID: 1QON. The MolDock Score (GRID) scoring function was used with a Grid resolution of 0.30 Å and 5 Å radius encompassing the entire connection cavity. The MolDock SE algorithm was used for the docking with a number of runs equal to 10, 1500 max interactions, and max population size equal to 50. The maximum evaluation of 300 steps, with neighbor distance factor of 1 and an energy threshold of 100, was used during the molecular docking simulation.

#### 3.6.2. MD Simulations

The charges of the methyl chavicol, linalool, 1,8-cineole and limonene atoms were calculated HF/6-31G*. Ligand parameters were constructed using GAFF and the proteins were described by the ff14SB force field in all simulations. The protonation of protein residues was evaluated using PROPKA server.

Each system was solvated in an octahedron periodic box with a 12-Å cutting radius in all directions from the solute (Waterp-TIP3P). An adequate number of counterions were added to neutralize the partial charge of the systems.

The MD simulations were performed using the Amber 16 software [61]. The minimization of system energy occurred in three steps. In the first step, 2000 cycles were executed using the steepest descent method and conjugate gradient algorithm, applying a harmonic force constant of 50 kcal·mol^−1^·Å^−2^ to the solute. In the second step, the harmonic force constant applied to the solute was 25 kcal·mol^−1^·Å^−2^ and 1000 more cycles were run using the steepest descent method and conjugate gradient algorithm. In the last step, the constraints were removed and 1000 cycles were run using steepest descent method and conjugate gradient algorithm.

To increase the system temperature from 0 to 300 k, 900 ps simulations were run. Warming up was carried out in three steps. In the first step, the solute was constrained with a harmonic force constant of 25 kcal·mol^−1^·Å^−2^, thus, only the solvent and counterions were free to move. In the following two steps, the harmonic force constant was removed. To balance the complexes, 2 ns simulations were run at constant temperature and without restrictions. Then, for each complex, 100 ns of MD simulation with NVT ensemble were generated.

#### 3.6.3. Free Energy Calculations

The free energy calculations were performed using MM-GBSA method [62]. The free energy was calculated as follows:

ΔG_bind_ = ΔH − TΔS ≈ ΔE_MM_ + ΔG_solv_ − TΔS (1)

where ΔG_bind_ is the free energy of the complex, resulting from the sum of the molecular mechanics energy (ΔE_MM_), desolvation free energy (ΔG_solv_), and entropy (−TΔS).

ΔE_MM_ = ΔE_internal_ + ΔE_electrostatic_ + ΔE_vdW_ (2)

The molecular mechanics energy of the gas phase (ΔE_MM_) can be described by the sum of the internal energy contributions (ΔE_internal_); sum of the connection, angle, and dihedral energies; electrostatic contributions (ΔE_electrostatic_); and van der Waals terms (ΔE_vdW_).

ΔG_solv_ = ΔG_GB_ + ΔG_nonpol_ (3)

The desolvation free energy (ΔG_solv_) is the sum of the polar (ΔG_GB_) and nonpolar (ΔG_nonpol_) contributions. The polar desolvation term was calculated using the implicit generalized born (GB) approach.

### 3.7. Statistical Analysis

Mortality data from replicates of each concentration were grouped and presented as mean ± standard deviation. The values of LC_50_, LC_90_ and 95% confidence intervals (CI) of upper and lower confidence levels were calculated from probit regression analysis using the software GraphicPad Prism 8. Larval mortality was corrected using the formula by Abbott [63], when mortality of the control group H_2_O varied between 5–20%. A one-way ANOVA statistical test was also performed, followed by a Tukey post-test. The statistical difference was considered significant when *p* < 0.05.

## 4. Conclusions

Chemical characterization of *O. basilicum* var. *minimum* essential oil extracted by stem distillation revealed the presence of hydrocarbon monoterpenes, oxygenated monoterpenes, hydrocarbon sesquiterpenes, oxygenated sesquiterpenes, with methyl chavicol, linalool, 1,8-cineole and limonene as the major compounds. In addition, the essential oil of *O. basilicum* var. *minimum* showed larvicidal action against *Ae. aegypti* larvae and the major compounds were able to interact with the binding cavity of the target enzyme acetylcholinesterase (AChE), indicating a potential ecological alternative for the control of larvae of this mosquito.

## Figures and Tables

**Table 1:** Chemical composition of *O. basilicum* var. *minimum* essential oil.

No.	RI_L_	RI_C_	Compound	Molecular Formula	Concentration (%)
1	969	967	Sabinene	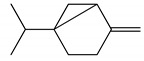	0.12
2	974	974	β-Pinene	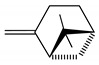	0.41
3	988	984	Myrcene	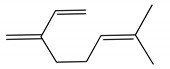	0.7
4	1024	1031	Limonene	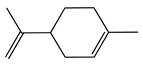	9.5
5	1026	1034	1,8-cineole	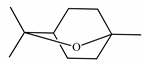	14.23
6	1044	1050	(*E*)-β-Ocimene	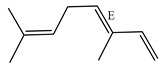	1.28
7	1054	1054	γ-Terpinene	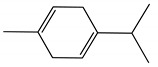	0.13
8	1095	1104	Linalool	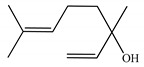	24.51
9	1141	1146	Camphor	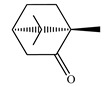	0.59
10	1174	1180	Terpinen-4-ol	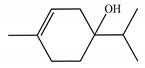	2.1
11	1195	1204	Methyl chavicol	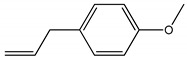	37.41
12	1239	1244	Carvone	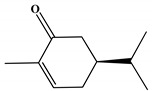	2.8
13	1247	1251	Chavicol	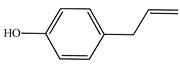	0.12
14	1289	1289	Thymol	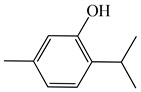	0.05
15	1335	1329	δ-Elemene	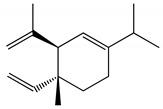	0.02
16	1389	1387	β-Elemene	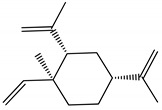	0.39
17	1417	1417	(*E*)-Caryophyllene	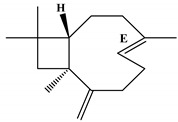	0.85
18	1432	1430	trans-α-Bergamotene	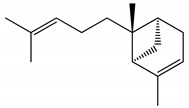	0.05
19	1454	1481	(*E*)-β-Farnesene	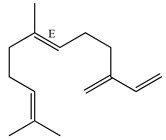	1.96
20	1484	1478	Germacrene D	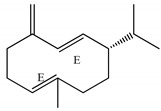	0.12
21	1489	1486	β-Selinene	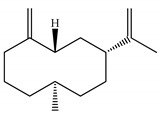	1.15
22	1493	1509	(*E*)-Muurola-4(14),5-diene	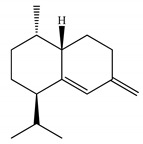	0.09
23	1498	1493	α-Selinene	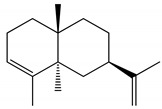	0.95
24	1638	1640	*epi*-α-cadinol	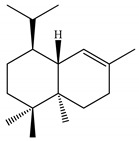	0.12
			Hydrocarbon monoterpenes		12.14
			Oxygenated monoterpenes		81.69
			Hydrocarbon sesquiterpenes		5.58
			Oxygenated sesquiterpenes		0.12
			Others compounds		0.12
			Total identified (%)		99.65

RI_C_: Retention Index (on DB-5MS column); RI_L_: literature retention index [22].

**Table 2:** Larvicidal activity of some essential oils of the genus *Ocimum* against *Ae. aegypti* larvae.

Specie	Plant Part	Plant Origin	LC_50_ (µg/mL)	Reference
*O. basilicum* var. *minimum*	Aerial	Brazilian Amazon	69.91 (61.89–78.58)	This work
*O. americanum*	Leaves	Northeast of Brazil	67.00	[38]
*O. basilicum*	Leaves	Pakistan	75.35 (53.21–108.08)	[39]
*O. campechianum*	Leaves	Northeast of Brazil	81.45	[40]
*O. carnosum*	Inflorescences	Northeast of Brazil	109.49	[40]
*O. gratissimum*	Aerial	Northeast of Brazil	60.00	[38]
*O. sanctun*	NM	India	92.42	[41]
*O. suave*	Leaves	Ethiopia	29.80 (23.5–35.0)	[42]

NM = Not mentioned.

## Data Availability

Not applicable.
